# Endochondral ossification pathway genes and postmenopausal osteoporosis: Association and specific allele related serum bone sialoprotein levels in Han Chinese

**DOI:** 10.1038/srep16783

**Published:** 2015-11-16

**Authors:** Yunzhi Zhang, Haiyan Liu, Chen Zhang, Tianxiao Zhang, Bo Zhang, Lu Li, Gang Chen, Dongke Fu, KunZheng Wang

**Affiliations:** 1 The First Department of Orthopedics, the Second Affiliated Hospital, School of Medicine, Xi’an Jiaotong University, Xi’an, Shaanxi, China; 2 Zhang’s Orthopaedic Hospital, Taizhou, Zhejiang, China; 3 Division of Biology & Biomedical Sciences, Washington University in Saint Louis, MO, USA; 4 School of Life Science and Technology, Xi’an Jiaotong University, Xi’an, Shaanxi, China; 5 Key Laboratory of Environment and Genes Related to Diseases, Ministry of Education, Xi’an, Shaanxi, China; 6 Key Laboratory of National Ministry of Health for Forensic Sciences, School of Medicine & Forensics, Xi’an Jiaotong University, Xi’an, Shaanxi, China

## Abstract

Osteoporosis is a systemic skeletal disorder characterized by reduced bone mineral density (BMD) and disrupted bone architecture, predisposing the patient to increased fracture risk. Evidence from early genetic epidemiological studies has indicated a major role for genetics in the development of osteoporosis and the variation in BMD. In this study, we focused on two key genes in the endochondral ossification pathway, *IBSP* and *PTHLH*. Over 9,000 postmenopausal Han Chinese women were recruited, and 54 SNPs were genotyped. Two significant SNPs within *IBSP*, rs1054627 and rs17013181, were associated with BMD and postmenopausal osteoporosis by the two-stage strategy, and rs17013181 was also significantly associated with serum IBSP levels. Moreover, one haplotype (rs12425376-rs10843047-rs42294) covering the 5’ end of *PTHLH* was associated with postmenopausal osteoporosis. Our results provide evidence for the association of these two key endochondral ossification pathway genes with BMD and osteoporosis in postmenopausal Han Chinese women. Combined with previous findings, we provide evidence that a particular SNP in *IBSP* has an allele-specific effect on mRNA levels, which would, in turn, reflect serum IBSP levels.

Osteoporosis (OP) is a systemic skeletal disorder characterized by reduced bone mineral density (BMD) and disrupted bone architecture, predisposing the patient to increased fracture risk^1^. Advanced age and gender are two important environmental factors for osteoporosis^2^,^3^,^4^, making osteoporosis a common bone disorder among postmenopausal women^1^,^4^. According to the surgeon general’s report on bone health and osteoporosis, approximately 4 out of 10 Caucasian women 50 years or older would experience osteoporosis-related fracture in their lifetime^4^. As a chronic condition, OP imposes a significant financial burden in women after menopause^5^,^6^.

OP and BMD are highly heritable^7^,^8^,^9^. An early twin study found that the heritability of bone mass was approximately 90% in the lumbar spine and 70% in the femoral neck^7^. Subsequent genetic association mapping studies, including a genome-wide association study (GWAS), identified several susceptibility genes that confer risk of OP and BMD^10^,^11^,^12^. Currently, more than 60 susceptible loci have been identified to be associated with BMD or OP^13^. Despite these attempts of association mapping based on common SNPs, the percentage of phenotypic variance explained by all these loci is still modest^13^,^14^. In addition, molecular and biological studies have lagged behind the genetic epidemiological studies. The biological mechanisms of over 30 BMD GWAS loci are still unclear^13^. Identifying specific susceptibility genes for BMD and OP can help reveal the underlying pathological mechanisms of OP, and in turn, inform the diagnosis, prevention, treatment strategy and novel drug development of this complex disorder.

Most bones form through the endochondral ossification, whereby the embryonic cartilaginous model gradually mineralizes and is replaced by bone^15^. As an essential process of skeletal development, it is systematically controlled by multiple regulatory factors. Genetic analysis incorporating the knowledge of biological pathways has identified gene clusters in the endochondral ossification pathway^16^. Several key factors of this pathway are associated with BMD or OP, including *IBSP*^17^,^18^,^19^, *PTH*^20^, *RUNX2*^21^,^22^, *SOX6*^23^ and *SP7*^24^. Previous studies have shown that *IBSP* is significantly associated with BMD in Americans^17^ and Australians^24^. Furthermore, unidirectional allele-specific regulation (ASE) of *IBSP* has been reported in human bone samples, with lower expression of the G allele compared to the A allele for SNP rs17013181^17^. However, it is still unclear whether the association of *IBSP* with BMD exists in a genetically independent Han Chinese population, and whether a similar allele-specific pattern identified at the RNA level would reflect serum protein levels.

To comprehensively investigate the susceptibility of genes involved in the endochondral ossification pathway, we examined the relationship between the two key genes *IBSP* and *PTHLH* and the diagnosis status of OP in over 9,000 postmenopausal women from the Han Chinese population. To determine whether the significant variants of OP are related to serum integrin-binding sialoprotein (IBSP, encoded by gene *IBSP*) levels, we measured IBSP serum levels in all of the samples.

## Materials and Methods

### Subjects

Two separate datasets were included in this study, and a two-stage approach was utilized for the discovery of single marker analyses. Subjects consisting of 1,425 women (aged 48–70 years) with primary postmenopausal osteoporosis and 3,557 healthy age-matched women (aged 48–70 years) were considered part of the discovery set and recruited from the Second Affiliated Hospital of Xi’an Jiaotong University and Xi’an Honghui Hospital. Subjects consisting of 1,039 women (aged 48–72 years) with primary postmenopausal osteoporosis and 3,032 healthy age-matched women (aged 48–72 years) were considered the replication set and enrolled from Taizhou Zhang’s Orthopaedic Hospital. Two-stage subjects recruited in this study were random genetically unrelated Han Chinese postmenopausal women from the city of Xi’an in Shaanxi Province and Taizhou in Zhejiang province with no migration history within the previous three generations. None of the subjects had a history of taking medication to treat osteoporosis, and subjects with diseases or medications known to affect bone metabolism were excluded from the controls. Given the possibility of osteoporosis resulting from obesity and the overall level of Asian body mass index (BMI), subjects with a BMI ≥ 27 were excluded from the study. The demographic information of the study subjects is shown in Table 1. This study was performed in accordance with the ethical guidelines of the Declaration of Helsinki (version 2002) and was approved by the Medical Ethics Committee of Xi’an Jiaotong University. All participants completed written informed consent forms.

### SNP selection and genotyping

All SNPs with minor allele frequencies (MAF) ≥ 0.01 were searched for between 15 kb upstream and 15 kb downstream of the *IBSP* and *PTHLH* genes in the HapMap HCB database by Haploview v4.2^25^. A total of 27 SNPs in the *IBSP* gene and 24 SNPs in the *PTHLH* gene were identified. Three additional SNPs in the *IBSP* gene were included from previous reports^17^,^24^. Based on the above criteria, 54 SNPs were included in further analyses (Supplementary Table S1). Peripheral blood was drawn from a vein into a sterile tube containing ethylenediamine tetraacetic acid (EDTA). Genomic DNA was extracted from peripheral blood leukocytes according to the manufacturer’s protocol (Genomic DNA kit, Axygen Scientific Inc., California, USA). DNA was stored at −20 °C for SNP analysis. Genotyping was performed for all SNPs using the MassARRAY platform (Sequenom, San Diego, California, USA). Briefly, SNPs were genotyped using high-throughput, matrix-assisted laser desorption ionization–time-of-flight (MALDI–TOF) mass spectrometry. The resulting spectra were processed using Typer Analyzer software (Sequenom), and genotype data were generated from the samples. As the final genotype call rate of each SNP was greater than 98% and the overall genotyping call rate was 99.6%, the reliability of further statistical analysis was ensured.

### Bone mineral density and serum bone turnover marker measurements

Dual-energy X-Ray absorptiometry (Lunar Expert 1313, Lunar Corp., USA) was used to measure BMD at the lumbar spine (L2-4) and femoral neck. Bone mineral density was determined according to standard Lunar protocols. Osteoporosis was defined according to the conventional World Health Organization (WHO) definition. Participants with a T score < −1.0 SD were classified as having low bone mass, and those with a T score > −1.0 SD were classified as normal. The anthropometric baseline data of all subjects were obtained through measurements and a questionnaire.

Peripheral venous blood was collected from each participant in the morning after a 12-hour fast according to the Declaration of Helsinki guidelines. Serum bone alkaline phosphatase (BALP) levels, osteocalcin (OST) levels, carboxyl-terminal telopeptide of type I collagen (CTX) levels, osteoprotegerin (OPG) levels, soluble receptor activator of nuclear factor-JB ligand (sRANKL) levels and bone sialoprotein levels (BSP) were measured using enzyme-linked immunosorbent assay (ELISA) kits (Westtang Biotech Inc., Shanghai, China). The minimal detection limit and the interassay and intra-assay coefficients of variation are presented for every marker in Supplemental Table S2. The summary of BMD and serum bone turnover marker measurements are shown in Table 1.

### Statistical analysis

A Hardy Weinberg Equilibrium (HWE) test and single marker association analysis was performed for sample sets of both stages by Plink^26^. OP diagnosis was considered the main phenotype in single marker based analyses. BMD measurements of the lumbar spine and femoral neck were analyzed as two quantitative traits by fitting a linear model. Age and BMI were included as covariates when conducting single marker based analyses. Haplotype-based analyses were also performed using Plink^26^. Linkage disequilibrium (LD) blocks were constructed and plotted using Haploview^25^. Bonferroni correction was applied for both the discovery and replication stages. SNPs that passed the Bonferroni corrected *P* value threshold (0.05/54 ≈ 0.0009) in the discovery stage were genotyped in the replication sample sets. SNPs with significant hits by LD (r^2^ > 0.1) in the discovery stage were included in the replication stage. In addition to the single marker based analyses, a conditioning model was fitted to test whether the significant SNPs identified in the single marker model were independent in the replication sample set. Furthermore, the association of serum IBSP level and genetic markers was analyzed by fitting a linear model using Plink^26^. All SNPs included in the replication stage were analyzed in combined sample sets. When fitting the linear model, age, BMI and sample site were considered covariates. A conditioning model was also fitted with the combined sample sets to test whether the effects of significant SNPs were independent. Bar plots were generated with R to visualize the association of SNP genotypes and quantitative traits^27^. Descriptive analyses were also conducted in R. Both Polyphen2^27^ and SIFT^28^ were used to predict the functional significance of significant non-synonymous SNPs identified in this study.

## Results

### Genetic association mapping of postmenopausal osteoporosis

**Figure 1:**
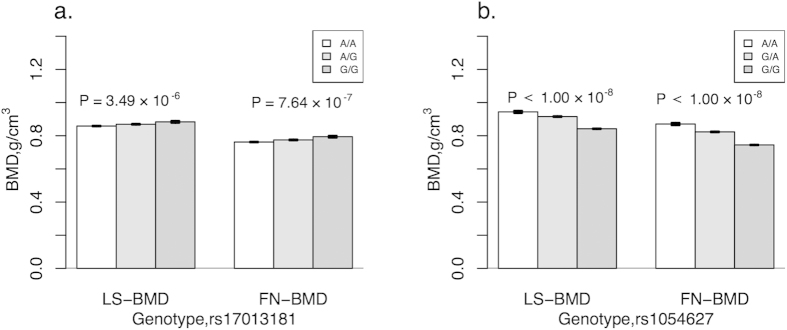
Association of bone mineral density and significant SNPs for postmenopausal osteoporosis. (**a**) SNP rs17013181; (**b**) SNP rs1054627. LS-BMD stands for BMD of lumbar spine. FN-BMD stands for BMD of femoral neck. The *P* values of association analyses in combine sample set were indicated for each BMD group.

The results of HWE and MAF are shown in Supplemental Table S1. All 54 SNPs passed the HWE test in the discovery stage. Two SNPs within the *IBSP* gene (rs1054627, *P* = 1.00 × 10^−4^; rs17013181, *P* = 4.99 × 10^−5^) passed the Bonferroni correction *P* value threshold in the discovery stage. We genotyped these 2 SNPs plus 11 additional SNPs in the same LD clusters in an independent analysis of the replication stage. The significance of both SNPs was successfully replicated in the second stage (rs1054627, *P* = 0.0001; rs17013181, *P* = 0.0003 with Bonferroni correction threshold of 0.05/13 ≈ 0.0038). After conditioning on SNP rs1054627, the significance of SNP rs17013181 was maintained (*P* = 0.0015). We did not identify any other SNPs with independent effects through single marker based analyses. The results of single marker based analyses of the 13 markers included in the replication stage are summarized in Table 2. We also performed association mapping analysis based on two BMD measurements (lumbar spine and femoral neck). The full results of association mapping for OP and BMD based on the discovery sample set are shown in Supplemental Table S3. The results of association mapping for BMD based on the replication sample set are presented in Supplemental Table S4. We confirmed the significance of both SNPs (rs1054627 and rs17013181) that we identified to be associated with OP in both stages in association analyses based on the BMD of the combined datasets (Fig. 1). As shown in Supplemental Figure S1 and S2, we constructed 18 LD blocks for the two genes (*IBSP* and *PTHLH*) based on the discovery sample set data. Haplotype analyses based on the discovery sample set data are summarized in Supplemental Table S5. We identified some significant haplotypes in the *IBSP* gene (rs6828578-rs958848, *P* = 2.98 × 10^−6^; rs4693878-rs1054627, *P* = 1.48 × 10^−7^; rs13144371-rs17013181, *P* = 3.26 × 10^−7^) and the *PTHLH* gene (rs12425376-rs10843047-rs42294, *P* = 5.75 × 10^−6^).

### Association of serum IBSP levels and IBSP gene SNPs

**Figure 2:**
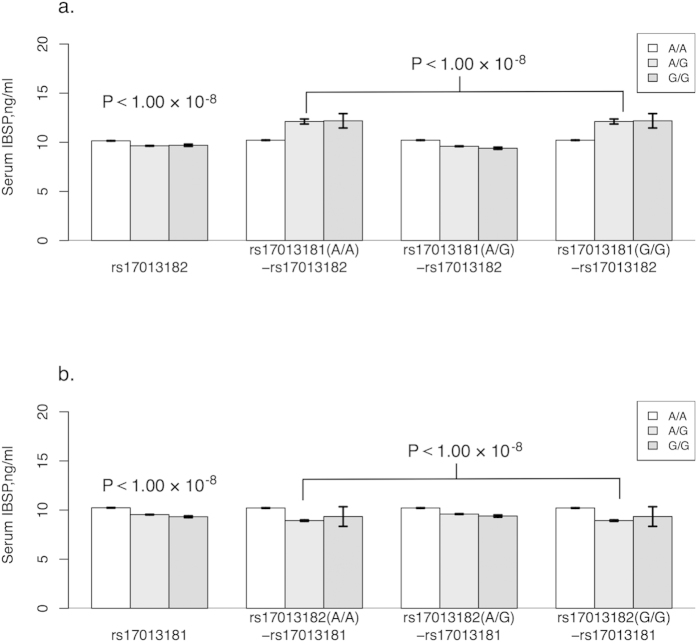
Association of rs17013181 and rs rs17013182 with serum IBSP level. (**a**) From left to right, rs17013182, rs17013182 conditioned on genotype A/A of rs17013181, rs17013182 conditioned on genotype A/G of rs17013181, rs17013182 conditioned on genotype G/G of rs17013181. (**b**) From left to right, rs17013181, rs17013181 conditioned on genotype A/A of rs17013182, rs17013181 conditioned on genotype A/G of rs17013182, rs17013181 conditioned on genotype G/G of rs17013182. The *P* values of association analyses in combine sample set were indicated for each group.

We determined that several SNPs in the *IBSP* gene were significantly associated with serum IBSP levels in the combined sample set. However, after conditioning on the two most significant SNPs (rs17013181 and rs17013182), none of the other SNPs showed significance (Table 3). Considering the strong LD between rs17013181 and rs17013182 (*r*^2^ = 0.83), we explored the relationship between serum IBSP levels and the combined genotype of the two SNPs (Fig. 2). After conditioning on the effects of rs17013181, we observed that the direction of the effects of rs17013182 was reversed (Table 3 and Fig. 2).

## Discussion

To our knowledge, this is the first large-scale genetic association study of *IBSP* and *PTHLH* genes with BMD, OP and serum IBSP levels in a Han Chinese population. Our results provide evidence for the association of two key endochondral ossification pathway genes with OP and BMD in postmenopausal Han Chinese women. Two SNPs with independent effects and some haplotypes in both *IBSP* and *PTHLH* genes were significantly associated with an OP diagnosis. We confirmed the effects of the two SNPs in analyses based on quantitative traits (BMD). Moreover, we also found evidence of an association between *IBSP* SNPs and serum IBSP levels.

Integrin-binding sialoprotein is an approximately 70 kDa acidic glycoprotein encoded by the human *IBSP* gene^29^. It belongs to the Small Integrin Binding Ligand N-linked Glycoprotein (SIBLING) gene family^30^. Previous studies have reported that IBSP is highly expressed in hypertrophic chondrocytes, which are at the center of endochondral ossification, and *IBSP* knockout mice have problems in bone resorption and mineralization^31^. In our study, we identified two non-synonymous SNPs within the *IBSP* gene (rs1701318 and rs1054627) that were significantly associated with the diagnosis of OP and variation in BMD. Conditioning analyses indicated that the effects of the two SNPs were independent. Previous reports have shown a significant association between SNP rs1054627 and BMD in populations of different ethnic groups. GWAS of Icelanders and replication samples of Europeans found this SNP to be significantly associated with femoral neck BMD^32^. It was also found to be significantly associated with femoral neck BMD in premenopausal European and African-American women^17^. In an extended genome-wide association study of bone mineral density based on 15,000 European individuals, rs1054627 in IBSP was significantly associated with bone density^18^,^19^. Among these previous studies, the directions of the effects were similar regardless of the ethnicity of the study samples. Although this SNP was predicted with limited functional significance by Polyphen2 (0.206) and SIFT (0.67), it might still alter protein structure and/or function because it changes the non-hydrophilic Gly residue to Glu, which is a hydrophilic residue^17^. Interestingly, this SNP was not associated with serum IBSP levels (*P* = 0.3134), which suggests that it did not affect *IBSP* expression level. Therefore, the effects of this SNP on OP diagnosis or BMD variation may solely come from the changes in protein structure. The other significant SNP, rs17013181, was also a non-synonymous SNP located in exon 7 of the *IBSP* gene. To our knowledge, this SNP have never been reported to be associated with OP or BMD diagnosis. In an earlier study^17^, researchers examined 52 human bone samples from the femoral neck during surgical hip replacement, and observed the allele-specific regulation of gene expression (ASE) for rs17013181. They reported that the minor allele G was expressed at lower levels than the ancestral allele A. In our study, we detected the association of rs17013181 with serum IBSP levels and observed a similar pattern (Table 3). The minor allele G was associated with lower serum IBSP levels (β = −0.6079, *P* = 4.51 × 10^−59^). Our findings suggest that the ASE pattern of *IBSP* is reflected at the protein level. Similar to SNP rs1054627, this SNP was predicted to have limited functional significance by Polyphen2 (0) and SIFT (0.36). However, this SNP can still affect OP onset and development and BMD variation by altering *IBSP* expression levels. The rs17013182 SNP in strong LD with rs17013181 was also significantly associated with serum IBSP levels (Table 3). Unlike rs17013181, this SNP only associated with serum IBSP levels, and not with the disease phenotype (Neither OP diagnosis nor BMD).

*PTHLH* is the protein-coding gene of parathyroid hormone-like related protein, which is a member of the parathyroid hormone family^33^. Parathyroid hormone (PTH) is a key regulator of calcium metabolism and contributes to skeletal development by regulating chondrocyte proliferation and differentiation during early bone growth^34^. Previous genetic studies have linked other PTH genes with age-related degenerative changes in lumbar spine^35^ and BMD^20^. In this study, we identified a three-SNP haplotype (rs12425376-rs10843047-rs42294, *P* = 5.75 × 10^−6^) that was significantly associated with the diagnosis of OP. The three SNPs were located near the 5′ end of the *PTHLH* gene. The functional relationship of the genomic region covered by this haplotype is unknown. However, this haplotype covers a transcription factor Chromatin Immunoprecipitation-Sequencing (ChIP-Seq) region. Several genes bind to this region, including *EZH2, YY1, TBP, ESR1, MAX, REST, USF1, SIN3AK20, ZNF143, SP2, CEBPB* and *SIN3A*^36^. Among these genes, *ESR1* and *EZH2* have been implicated in bone maturation^11^,^37^,^38^. However, further study is required to explain the significance of these association signals.

Our study has several strengths. The large sample size of our study (>9,000) ensured the statistical power to detect modest effects of SNPs. In addition, we utilized a two-stage study design (discovery stage and replication stage). With this study design, SNPs that were identified as significant in both stages were unlikely to be false positives. Unlike previous studies that focused on the relationship between genetic polymorphisms and disease phenotypes, we measured serum IBSP levels to further investigate the potential effects of susceptibility SNPs on gene expression. However, several limitations should also be kept in mind. Possible population admixture and stratification could complicate our findings, although we have applied several strategies to reduce potential population stratification during the sample collection stage. Furthermore, our study consisted only of postmenopausal women, and from the perspective of epidemiology, this subject selection strategy impaired the representativeness of our study. We also failed to measure hormone levels in our subjects, which could confound our results. In addition, like most common SNP-based studies, we only included genetic variations with MAF ≥ 0.01 for association and therefore cannot detect the potential contribution of rare variants. Recent genetic epidemiology studies have indicated that rare variants play important roles in the onset and development of complex disorders^39^,^40^,^41^,^42^,^43^,^44^,^45^. In addition to the genetic variations in its encoding gene, serum IBSP levels can be affected by several other biological factors that cannot be effectively examined or controlled in association analyses between serum IBSP level and SNPs in the *IBSP* gene. This could potentially weaken the convincingness of our findings.

## Conclusions

In summary, our results provide further supportive evidence for the association of the *IBSP* and *PTHLH* genes with the diagnosis of OP and of the *IBSP* gene with variation of BMD in postmenopausal Han Chinese women. Combined with previous findings, we provided evidence that a particular SNP in *IBSP* has an allele-specific effect on the mRNA level, which would in turn, affect serum IBSP levels. Two SNPs in *IBSP,* rs1054627 and rs17013181, may confer risk of postmenopausal osteoporosis in Han Chinese women, and be useful in the informative assessment of the genetic risk for reduced BMD together with serum IBSP level. However, in view of multiple variants with small effects and the molecular basis of associations in relation to the complex network underlying bone remodeling and bone mass, further studies are required, including high density mapping and deep sequencing in different populations, to identify possible causal variants and determine the detailed mechanism of how these variants affect *IBSP* expression.

## Additional Information

**How to cite this article**: Zhang, Y. *et al.* Endochondral ossification pathway genes and postmenopausal osteoporosis: Association and specific allele related serum bone sialoprotein levels in Han Chinese. *Sci. Rep.* **5**, 16783; doi: 10.1038/srep16783 (2015).

## Supplementary Material

Supplementary Information

## Figures and Tables

**Table 1:** The summary of demographic information, measurements of BMD and bone turnover markers of study subjects.

	Discovery (N = 4,982)		Replication (N = 4,982)		
	Case (N = 1,425)	Control (N = 3,557)	Case (N = 1,039)	Control (N = 3,032)	All samples (N = 9,053)
Age, years (sd)	62.11 (11.31)	61.80 (6.30)	62.30 (6.87)	62.50 (6.93)	62.14 (6.59)
BMI, kg/m2 (sd)	23.42 (1.86)	23.53 (1.92)	23.06 (1.56)	22.98 (1.46)	23.27 (1.75)
menopause, years (sd)	11.31 (5.29)	11.20 (5.68)	10.76 (5.53)	10.63 (5.95)	10.98 (5.70)
LN-BMD, g/cm3 (sd)	0.71 (0.06)	0.90 (0.05)	0.74 (0.06)	0.93 (0.07)	0.86 (0.10)
FN-BMD, g/cm3 (sd)	0.60 (0.07)	0.81 (0.06)	0.63 (0.07)	0.84 (0.06)	0.77 (0.11)
IBSP, ng/ml (sd)	11.72 (1.89)	9.47 (1.51)	11.60 (1.97)	9.25 (1.66)	9.99 (1.97)
BALP, μg/l (sd)	26.34 (1.78)	21.57 (1.77)	26.14 (1.70)	21.33 (1.81)	22.76 (2.78)
CTX, μg/l (sd)	0.89 (0.12)	0.72 (0.16)	0.86 (0.12)	0.70 (0.16)	0.76 (0.17)
OST, ng/ml (sd)	12.33 (2.14)	9.13 (2.29)	12.26 (2.22)	9.09 (2.22)	9.98 (2.65)
OPG, ng/ml (sd)	8.83 (1.43)	10.46 (1.37)	8.61 (1.39)	9.99 (1.35)	9.83 (1.54)
SRANKL, pg/ml (sd)	6.84 (0.96)	9.78 (1.83)	6.81 (0.91)	9.64 (1.83)	8.93 (2.08)
OPG/SKL*1000 (sd)	1.32 (0.29)	1.11 (0.25)	1.29 (0.28)	1.07 (0.25)	1.15 (0.28)

BMI, body mass index; BMD, bone mineral density; LS, lumbar spine; FN, femoral neck; IBSP, serum integrin-binding sialoprotein; BALP, Serum bone alkaline phosphatase; CTX, carboxy-terminal telopeptide of type I collagen; OST, osteocalcin; OPG, osteoprotegerin; sRANKL, soluble receptor activator of nuclear factor-JB ligand.

**Table 2:** Results of the single marker based association analysis for the 13 SNPs within *IBSP* genotyped in the replication stage.

CHR	SNP	BP	A1	OR_1*	P_1*	OR_2**	P_2**	OR_3***	P_3***
4	rs958479	87800630	C	0.9507	0.2739	0.9761	0.6374	0.9871	0.8017
4	rs3805376	87800675	T	1.0350	0.4843	1.0410	0.4762	1.0600	0.3008
4	rs2616262	87802058	G	1.1270	0.0077	1.1030	0.0530	1.1260	0.0207
4	rs4693877	87803043	C	1.0270	0.6319	1.0440	0.5065	1.0790	0.2420
4	rs1870964	87803448	A	1.0580	0.3215	1.0260	0.6910	1.0610	0.3669
4	rs3805374	87803815	C	1.0600	0.2998	1.1160	0.1316	1.4410	2.52 × 10^−5^
4	rs1381965	87807752	C	0.9672	0.4601	1.0650	0.2305	1.3770	3.51 × 10^−5^
4	rs4693878	87811379	T	1.0350	0.4466	1.0390	0.4531	1.4690	4.79 × 10^−5^
**4**	**rs1054627**	**87811540**	**A**	**0.7682**	**0.0001**	**0.7447**	**0.0001**	**NA**	**NA**
4	rs13144371	87811594	A	1.0220	0.6322	1.0300	0.5651	1.0580	0.2740
**4**	**rs17013181**	**87811611**	**G**	**0.7860**	**4.99** **×** **10**^**−5**^	**0.7746**	**0.0003**	**0.7997**	**0.0015**
4	rs17013182	87811722	G	1.0480	0.4227	1.0720	0.3137	1.1080	0.1387
4	rs1054628	87811759	T	1.0410	0.4175	1.0640	0.2738	1.0840	0.1555

Significant SNPs were highlighted in bold.

^*^Odds ratio and *P* values for analysis based on the sample of discovery stage.

^**^Odds ratio and *P* values for analysis based on the sample of replication stage.

^***^Odds ratio and *P* values for analysis based on the sample of replication stage while conditioning on SNP rs1054627.

**Table 3:** Results of association analysis for serum IBSP level based on the data from combined sample set.

CHR	SNP	BP	A1	BETA_1*	*P*_1*	BETA_2**	*P*_2**	BETA_3***	*P*_3***
4	rs958479	87800630	C	−0.0034	0.9102	−0.0100	0.7351	−0.0113	0.7002
4	rs3805376	87800675	T	0.0272	0.4039	0.0287	0.3711	0.0269	0.4006
4	rs2616262	87802058	G	0.0685	0.0193	0.0718	0.0129	0.0720	0.0122
4	rs4693877	87803043	C	−0.0003	0.9942	0.0123	0.7335	0.0099	0.7831
4	rs1870964	87803448	A	0.0084	0.8221	0.0233	0.5267	0.0208	0.5694
4	rs3805374	87803815	C	0.0208	0.5957	0.0477	0.2171	0.0466	0.2262
4	rs1381965	87807752	C	−0.0011	0.9695	0.0303	0.3036	0.0347	0.2369
4	rs4693878	87811379	T	0.0003	0.9914	0.0382	0.1935	0.0415	0.1564
4	rs1054627	87811540	A	−0.1743	2.48 × 10^−5^	−0.1125	0.0060	−0.1060	0.0093
4	rs13144371	87811594	A	−0.2783	2.69 × 10^−21^	0.1052	0.0097	0.0939	0.0205
**4**	**rs17013181**	**87811611**	**G**	**−0.6079**	**4.51** **×** **10**^**−59**^	**NA**	**NA**	**NA**	**NA**
**4**	**rs17013182**	**87811722**	**G**	**−0.4170**	**1.43** **×** **10**^**−26**^	**0.8097**	**9.89** **×** **10**^**−20**^	**NA**	**NA**
4	rs1054628	87811759	T	−0.2797	7.54 × 10^−18^	0.1297	0.0027	−0.0481	0.3134

Significant SNPs were highlighted in bold.

^*^Linear regression coefficient and *P* values based on the data from combined sample set.

^**^Linear regression coefficient and *P* values after conditioning on SNP rs17013181.

^***^Linear regression coefficient and *P* values after conditioning on SNP rs17013181 and rs17013182.
