# Supplementary material for: Endochondral ossification pathway genes and postmenopausal osteoporosis: Association and specific allele related serum bone sialoprotein levels in Han Chinese
Source: Sci Rep. 2015 Nov 16;5:16783. doi: 10.1038/srep16783 (PMC4645187; doi:10.1038/srep16783)
Supplement: Supplementary Information [file srep16783-s1.pdf]

Title: Endochondral ossification pathway genes and postmenopausal osteoporosis: Association and specific allele related serum bone sialoprotein levels in Han Chinese

Author names and affiliations: Yunzhi Zhang [a,b], Haiyan Liu [b], Chen Zhang [a], Tianxiao Zhang [c], Bo Zhang [d], Lu Li [e,f], Gang Chen [e,f] , Dongke Fu [e,f] and KunZheng Wang [a]

[a] The First Department of Orthopedics, the Second Affiliated Hospital, School of Medicine, Xi'an Jiaotong University, Xi’an, Shaanxi, China;

[b] Zhang’s Orthopaedic Hospital, Taizhou, Zhejiang, China;

[c] Division of Biology & Biomedical Sciences, Washington University in Saint Louis, MO, USA;

[d] School of Life Science and Technology, Xi'an Jiaotong University, Xi'an, Shaanxi, China;

[e] Key Laboratory of Environment and Genes Related to Diseases, Ministry of Education, Xi'an, Shaanxi, China;

[f] Key Laboratory of National Ministry of Health for Forensic Sciences, School of Medicine & Forensics, Xi’an Jiaotong University, Xi’an, Shaanxi, China.

Corresponding Author:

Kunzheng Wang, Ph.D., M.D., The First Department of Orthopedics, the Second Affiliated Hospital, School of Medicine, Xi'an Jiaotong University, 157 Xiwu road, Xi’an, China, 710061. Tel: +86-29-87679425, Fax: +86-29-87679323, E-mail: kunzh_wang@163.com

Supplemental Table S1. Summarized information of the 54 selected SNPs.

CHR	SNP	A1	A2	POS	FUNCTION	GENE	MAF_1*	HWE_1*	MAF_2**	HWE_2**
4	rs13136331	T	C	88707081	unknown	*IBSP*	0.201	0.875	NA	NA
4	rs17013148	A	G	88707290	unknown	*IBSP*	0.087	0.915	NA	NA
4	rs6828578	T	C	88707553	unknown	*IBSP*	0.125	1.000	NA	NA
4	rs958848	G	C	88709269	unknown	*IBSP*	0.115	0.802	NA	NA
4	rs17761748	C	A	88709783	unknown	*IBSP*	0.148	0.947	NA	NA
4	rs2126650	T	C	88710587	unknown	*IBSP*	0.155	0.898	NA	NA
4	rs11097170	T	A	88711266	unknown	*IBSP*	0.338	0.970	NA	NA
4	rs1463109	C	G	88714718	unknown	*IBSP*	0.330	0.970	NA	NA
4	rs1463108	A	G	88714805	unknown	*IBSP*	0.324	1.000	NA	NA
4	rs7672820	C	G	88715259	unknown	*IBSP*	0.079	0.907	NA	NA
4	rs7688887	T	C	88715298	unknown	*IBSP*	0.102	0.717	NA	NA
4	rs13137552	C	T	88715324	unknown	*IBSP*	0.168	0.951	NA	NA
4	rs17013159	G	C	88715821	unknown	*IBSP*	0.146	1.000	NA	NA
4	rs958479	C	A	88721782	intron	*IBSP*	0.365	0.914	0.440	0.941
4	rs3805376	T	C	88721827	intron	*IBSP*	0.274	1.000	0.281	1.000
4	rs2616262	G	T	88723210	intron	*IBSP*	0.426	0.973	0.460	1.000
4	rs4693877	C	G	88724195	intron	*IBSP*	0.199	1.000	0.192	1.000
4	rs1870964	A	C	88724600	intron	*IBSP*	0.188	0.868	0.188	1.000
4	rs3805374	C	T	88724967	intron	*IBSP*	0.193	1.000	0.136	0.937
4	rs1381965	C	A	88728904	intron	*IBSP*	0.402	0.835	0.387	0.908
4	rs4693878	T	C	88732531	coding-synon	*IBSP*	0.371	0.971	0.457	1.000
4	rs1054627	A	G	88732692	missense	*IBSP*	0.150	0.950	0.141	1.000
4	rs13144371	A	G	88732746	missense	*IBSP*	0.407	0.835	0.479	0.942
4	rs17013181	G	A	88732763	missense	*IBSP*	0.185	0.709	0.178	1.000
4	rs17013182	G	A	88732874	missense	*IBSP*	0.171	0.905	0.158	1.000
4	rs1054628	T	C	88732911	missense	*IBSP*	0.279	0.834	0.274	0.927
4	rs1054629	T	A	88732918	missense	*IBSP*	0.155	0.850	NA	NA
4	rs7681895	T	C	88742318	near-gene-5	*IBSP*	0.395	0.916	NA	NA
4	rs13128741	T	C	88743279	intron	*IBSP*	0.069	0.691	NA	NA
4	rs13117929	G	A	88746734	intron	*IBSP*	0.120	0.813	NA	NA
12	rs1861912	T	C	28099332	unknown	*PTHLH*	0.333	0.821	NA	NA
12	rs1861911	T	G	28099396	unknown	*PTHLH*	0.332	0.850	NA	NA
12	rs10843031	C	T	28099496	unknown	*PTHLH*	0.169	1.000	NA	NA
12	rs10492365	G	A	28100372	unknown	*PTHLH*	0.161	0.950	NA	NA
12	rs10843037	T	G	28103775	unknown	*PTHLH*	0.322	0.939	NA	NA
12	rs3906888	C	A	28104655	unknown	*PTHLH*	0.395	0.944	NA	NA
12	rs10843041	A	G	28110604	near-gene-3	*PTHLH*	0.149	0.841	NA	NA
12	rs6252	C	T	28111073	untranslated-3	*PTHLH*	0.152	1.000	NA	NA

12	rs10492364	T	C	28112256	intron	*PTHLH*	0.333	0.880	NA	NA
12	rs6248	G	A	28114816	intron,near-gene-3	*PTHLH*	0.145	0.892	NA	NA
12	rs6245	T	C	28116057	intron,untranslated-3	*PTHLH*	0.124	0.876	NA	NA
12	rs2796	T	C	28116111	intron,untranslated-3	*PTHLH*	0.154	0.899	NA	NA
12	rs805512	C	A	28118847	intron	*PTHLH*	0.324	0.878	NA	NA
12	rs997199	A	C	28119384	intron	*PTHLH*	0.151	0.894	NA	NA
12	rs997200	C	T	28119561	intron	*PTHLH*	0.435	1.000	NA	NA
12	rs3794275	A	C	28120465	intron	*PTHLH*	0.134	0.942	NA	NA
12	rs27633	G	T	28124305	intron,near-gene-5	*PTHLH*	0.343	0.882	NA	NA
12	rs39635	A	C	28126245	near-gene-5	*PTHLH*	0.341	1.000	NA	NA
12	rs1268693	G	A	28126939	near-gene-5	*PTHLH*	0.439	1.000	NA	NA
12	rs12425376	T	C	28128625	unknown	*PTHLH*	0.427	0.945	NA	NA
12	rs10843047	G	A	28132512	unknown	*PTHLH*	0.255	0.894	NA	NA
12	rs42294	G	T	28137869	unknown	*PTHLH*	0.246	0.928	NA	NA
12	rs11049257	C	T	28139005	unknown	*PTHLH*	0.145	0.891	NA	NA
12	rs33083	A	G	28139312	unknown	*PTHLH*	0.256	0.894	NA	NA

* Minor allele frequency and hardy weinburg equilibrium test *P* values in the discovery stage.

** Minor allele frequency and hardy weinburg equilibrium test *P* values in the replication stage.

Supplemental Table S2. The minimal detection limit and the interassay and intra-assay coefficients of variation for serum bone turnover markers.

Marker	Minimal detection limit	Interassay coefficients of variation (%)	Intra-assay coefficients of variation (%)
BALP	< 5 U/L	< 10	< 10
OST	< 0.15 ng/ml	< 12	< 12
CTX	< 30 pg/ml	< 10.6	< 10.6
BSP	< 15 pg/ml	< 10.2	< 10.2
OPG	< 0.5 pmol/L	< 8.9	< 8.9
sRANKL	< 12 pg/ml	< 9.2	< 9.2

Supplemental Table S3. Results of single marker based association analyses of 54 SNPs based on the discovery stage sample set.

CHR	SNP	BP	A1	OR*	P*	BETA_LS**	P_LS**	BETA_FN***	P_FN***
4	rs13136331	87785929	T	1.052	0.3576	-0.0009	0.6914	-0.0008	0.7609
4	rs17013148	87786138	A	1.054	0.5031	-0.0024	0.4642	-0.0019	0.5874
4	rs6828578	87786401	T	0.932	0.3037	0.0038	0.1676	0.0039	0.2000
4	rs958848	87788117	G	1.074	0.2969	-0.0018	0.5277	-0.0021	0.5020
4	rs17761748	87788631	C	1.063	0.3213	-0.0017	0.5165	-0.0021	0.4643
4	rs2126650	87789435	T	1.032	0.6032	-0.0008	0.7407	-0.0010	0.7074
4	rs11097170	87790114	T	1.034	0.4751	-0.0024	0.2263	-0.0026	0.2222
4	rs1463109	87793566	C	1.017	0.7234	-0.0018	0.3453	-0.0019	0.3737
4	rs1463108	87793653	A	1.047	0.3348	-0.0031	0.1195	-0.0032	0.1377
4	rs7672820	87794107	C	1.049	0.5556	-0.0008	0.8047	-0.0011	0.7630
4	rs7688887	87794146	T	0.966	0.6397	0.0030	0.3250	0.0038	0.2497
4	rs13137552	87794172	C	1.092	0.1325	-0.0023	0.3489	-0.0025	0.3549
4	rs17013159	87794669	G	0.909	0.1344	0.0093	0.0004	0.0102	0.0003
**4**	**rs958479**	**87800630**	**C**	**0.951**	**0.2739**	**0.0088**	**3.84E-06**	**0.0094**	**6.35E-06**
4	rs3805376	87800675	T	1.035	0.4843	0.0062	0.0025	0.0066	0.0032
4	rs2616262	87802058	G	1.127	0.0077	0.0042	0.0235	0.0045	0.0248
**4**	**rs4693877**	**87803043**	**C**	**1.027**	**0.6319**	**0.0137**	**2.31E-09**	**0.0145**	**6.71E-09**
**4**	**rs1870964**	**87803448**	**A**	**1.058**	**0.3215**	**0.0137**	**6.17E-09**	**0.0144**	**1.69E-08**
**4**	**rs3805374**	**87803815**	**C**	**1.060**	**0.2998**	**0.0261**	**1.77E-29**	**0.0273**	**2.35E-27**
**4**	**rs1381965**	**87807752**	**C**	**0.967**	**0.4601**	**0.0309**	**4.14E-63**	**0.0327**	**6.75E-60**
**4**	**rs4693878**	**87811379**	**T**	**1.035**	**0.4466**	**0.0311**	**9.96E-62**	**0.0327**	**7.28E-58**
**4**	**rs1054627**	**87811540**	**A**	**0.768**	**0.0001**	**0.0720**	**2.59E-186**	**0.0772**	**1.15E-180**
4	rs13144371	87811594	A	1.022	0.6322	0.0012	0.5115	0.0025	0.2271
**4**	**rs17013181**	**87811611**	**G**	**0.786**	**4.99E-05**	**0.0110**	**3.49E-06**	**0.0127**	**7.64E-07**
4	rs17013182	87811722	G	1.048	0.4227	0.0004	0.8736	0.0008	0.7607
4	rs1054628	87811759	T	1.041	0.4175	-0.0002	0.9109	-0.0003	0.9070
4	rs1054629	87811766	T	0.927	0.2256	0.0028	0.2733	0.0029	0.2924
4	rs7681895	87821166	T	0.965	0.4310	0.0011	0.5706	0.0010	0.6146
4	rs13128741	87822127	T	1.061	0.4898	-0.0010	0.7755	-0.0014	0.7272
4	rs13117929	87825582	G	0.960	0.5555	0.0011	0.7089	0.0014	0.6460
12	rs1861912	27946399	T	0.962	0.4166	0.0013	0.4920	0.0010	0.6264
12	rs1861911	27946463	T	1.026	0.5787	-0.0012	0.5442	-0.0017	0.4117
12	rs10843031	27946563	C	1.034	0.5707	-0.0019	0.4402	-0.0028	0.2940
12	rs10492365	27947439	G	1.046	0.4572	-0.0026	0.2952	-0.0035	0.2041
12	rs10843037	27950842	T	1.027	0.5761	-0.0008	0.6942	-0.0015	0.4974
12	rs3906888	27951722	C	1.027	0.5628	-0.0005	0.8082	-0.0009	0.6628
12	rs10843041	27957671	A	1.041	0.5180	-0.0025	0.3236	-0.0028	0.3233

12	rs6252	27958140	C	1.025	0.6901	-0.0019	0.4619	-0.0020	0.4642
12	rs10492364	27959323	T	0.978	0.6304	0.0010	0.6191	0.0007	0.7547
12	rs6248	27961883	G	1.042	0.5127	-0.0032	0.2175	-0.0037	0.1848
12	rs6245	27963124	T	1.062	0.3633	-0.0036	0.1967	-0.0041	0.1770
12	rs2796	27963178	T	0.963	0.5413	-0.0004	0.8857	-0.0004	0.8740
12	rs805512	27965914	C	1.040	0.4105	-0.0030	0.1267	-0.0033	0.1215
12	rs997199	27966451	A	1.073	0.2524	-0.0049	0.0580	-0.0048	0.0836
12	rs997200	27966628	C	0.980	0.6432	-0.0006	0.7566	-0.0006	0.7671
12	rs3794275	27967532	A	1.070	0.2972	-0.0022	0.4236	-0.0017	0.5518
12	rs27633	27971372	G	0.981	0.6776	0.0010	0.5985	0.0015	0.4635
12	rs39635	27973312	A	1.032	0.4942	-0.0010	0.5921	-0.0008	0.7071
12	rs1268693	27974006	G	1.033	0.4702	-0.0003	0.8499	-0.0002	0.9391
12	rs12425376	27975692	T	1.019	0.6811	-0.0011	0.5695	-0.0010	0.6259
12	rs10843047	27979579	G	1.023	0.6513	-0.0011	0.6137	-0.0010	0.6763
12	rs42294	27984936	G	0.966	0.5094	0.0016	0.4588	0.0018	0.4323
12	rs11049257	27986072	C	1.066	0.3086	-0.0023	0.3856	-0.0027	0.3443
12	rs33083	27986379	A	1.045	0.3849	-0.0011	0.6049	-0.0015	0.5189

Significant SNPs were highlighted in bold.

* Odds ratio and *P* values for analysis based on the sample of discovery stage.

**Linear regression coefficient and *P* values for analysis of bone mineral density of lumbar spine based on the sample of discovery stage.

*** Linear regression coefficient and *P* values for analysis of bone mineral density of femoral neck based on the sample of discovery stage.

Supplemental Table S4. Results of single marker based association analyses of 13 SNPs for BMD based on the replication stage sample set.

CHR	SNP	BP	A1	BETA_LS*	P_LS*	BETA_FN**	P_FN**
4	rs958479	87800630	C	0.0036	0.0791	0.0037	0.1032
4	rs3805376	87800675	T	0.0029	0.1986	0.0032	0.1935
4	rs2616262	87802058	G	0.0011	0.5903	0.0011	0.6091
4	rs4693877	87803043	C	0.0053	0.0427	0.0056	0.0496
4	rs1870964	87803448	A	0.0062	0.0179	0.0064	0.0245
**4**	**rs3805374**	**87803815**	**C**	**0.0271**	**8.76E-20**	**0.0287**	**7.83E-19**
**4**	**rs1381965**	**87807752**	**C**	**0.0228**	**1.42E-27**	**0.0251**	**3.20E-28**
**4**	**rs4693878**	**87811379**	**T**	**0.0263**	**3.82E-38**	**0.0295**	**1.68E-40**
**4**	**rs1054627**	**87811540**	**A**	**0.0608**	**1.97E-99**	**0.0705**	**1.17E-113**
4	rs13144371	87811594	A	0.0052	0.0109	0.0052	0.0203
**4**	**rs17013181**	**87811611**	**G**	**0.0151**	**1.64E-08**	**0.0186**	**1.63E-10**
4	rs17013182	87811722	G	0.0032	0.2539	0.0046	0.1310
4	rs1054628	87811759	T	0.0008	0.7348	0.0015	0.5440

Significant SNPs were highlighted in bold.

*Linear regression coefficient and *P* values for analysis of bone mineral density of lumbar spine based on the sample of replication stage.

** Linear regression coefficient and *P* values for analysis of bone mineral density of femoral neck based on the sample of replication stage.

Supplemental Table S5. Results of the haplotype-based association analyses using data from discovery sample set.

LOCUS	HAPLOTYPE	F_A	F_U	CHISQ	DF	P	SNPS	GENE
**H1**	**OMNIBUS**	**NA**	**NA**	**25.44**	**2**	**2.98E-06**	**rs6828578|rs958848**	**IBSP**
H1	TG	0.1166	0.112	0.4266	1	0.5137	rs6828578|rs958848	IBSP
H1	TC	0.0032	0.0152	25.24	1	5.07E-07	rs6828578|rs958848	IBSP
H1	CC	0.8802	0.8728	1.023	1	0.3118	rs6828578|rs958848	IBSP
H2	CT	0.1529	0.1466	0.6251	1	0.4292	rs17761748|rs2126650	IBSP
H2	AC	0.8471	0.8534	0.6251	1	0.4292	rs17761748|rs2126650	IBSP
H3	TCA	0.3321	0.3252	0.4376	1	0.5083	rs11097170|rs1463109|rs1463108	IBSP
H3	AGG	0.6679	0.6748	0.4376	1	0.5083	rs11097170|rs1463109|rs1463108	IBSP
H4	OMNIBUS	NA	NA	2.923	2	0.2319	rs17013159|rs958479	IBSP
H4	GC	0.1354	0.1487	2.897	1	0.08875	rs17013159|rs958479	IBSP
H4	CC	0.2215	0.2195	0.04588	1	0.8304	rs17013159|rs958479	IBSP
H4	CA	0.6431	0.6318	1.124	1	0.2892	rs17013159|rs958479	IBSP
H5	OMNIBUS	NA	NA	11.71	3	0.00844	rs2616262|rs4693877|rs1870964	IBSP
H5	GCA	0.191	0.1848	0.5156	1	0.4727	rs2616262|rs4693877|rs1870964	IBSP
H5	GCC	0.0095	0.01278	1.85	1	0.1738	rs2616262|rs4693877|rs1870964	IBSP
H5	GGC	0.2475	0.2207	8.226	1	0.004129	rs2616262|rs4693877|rs1870964	IBSP
H5	TGC	0.552	0.5817	7.302	1	0.006888	rs2616262|rs4693877|rs1870964	IBSP
H6	OMNIBUS	NA	NA	3.228	2	0.199	rs3805374|rs1381965	IBSP
H6	CC	0.1976	0.1895	0.8663	1	0.352	rs3805374|rs1381965	IBSP
H6	TC	0.1994	0.2149	2.964	1	0.08514	rs3805374|rs1381965	IBSP
H6	TA	0.603	0.5956	0.4677	1	0.494	rs3805374|rs1381965	IBSP
**H7**	**OMNIBUS**	**NA**	**NA**	**31.45**	**2**	**1.48E-07**	**rs4693878|rs1054627**	**IBSP**
H7	TA	0.1246	0.1579	17.87	1	2.36E-05	rs4693878|rs1054627	IBSP
H7	TG	0.2533	0.2114	20.58	1	5.71E-06	rs4693878|rs1054627	IBSP
H7	CG	0.6221	0.6307	0.6407	1	0.4235	rs4693878|rs1054627	IBSP
**H8**	**OMNIBUS**	**NA**	**NA**	**29.87**	**2**	**3.26E-07**	**rs13144371|rs17013181**	**IBSP**
H8	AG	0.1583	0.1942	17.45	1	2.95E-05	rs13144371|rs17013181	IBSP
H8	AA	0.2536	0.2122	20.01	1	7.70E-06	rs13144371|rs17013181	IBSP
H8	GA	0.5881	0.5936	0.2517	1	0.6159	rs13144371|rs17013181	IBSP
H9	OMNIBUS	NA	NA	0.8398	2	0.6571	rs17013182|rs1054628	IBSP
H9	GT	0.1738	0.1679	0.4996	1	0.4797	rs17013182|rs1054628	IBSP
H9	AT	0.1126	0.1094	0.2147	1	0.6431	rs17013182|rs1054628	IBSP
H9	AC	0.7136	0.7227	0.8357	1	0.3606	rs17013182|rs1054628	IBSP
H10	OMNIBUS	NA	NA	0.4197	2	0.8107	rs1861912|rs1861911|rs10843031	PTHLH
H10	TTC	0.1631	0.168	0.346	1	0.5564	rs1861912|rs1861911|rs10843031	PTHLH
H10	TTT	0.1667	0.1635	0.1465	1	0.7019	rs1861912|rs1861911|rs10843031	PTHLH
H10	CGT	0.6702	0.6685	0.027	1	0.8695	rs1861912|rs1861911|rs10843031	PTHLH

H11	OMNIBUS	NA	NA	0.4239	2	0.809	rs10492365|rs10843037	PTHLH
H11	GT	0.1623	0.1583	0.2417	1	0.623	rs10492365|rs10843037	PTHLH
H11	AT	0.1647	0.1621	0.1053	1	0.7455	rs10492365|rs10843037	PTHLH
H11	AG	0.673	0.6797	0.4115	1	0.5212	rs10492365|rs10843037	PTHLH
H12	OMNIBUS	NA	NA	0.4459	2	0.8002	rs3906888|rs10843041	PTHLH
H12	CA	0.1496	0.1462	0.1871	1	0.6654	rs3906888|rs10843041	PTHLH
H12	CG	0.251	0.2472	0.1527	1	0.696	rs3906888|rs10843041	PTHLH
H12	AG	0.5994	0.6066	0.4338	1	0.5101	rs3906888|rs10843041	PTHLH
H13	OMNIBUS	NA	NA	0.8945	3	0.8268	rs10492364|rs6248|rs6245	PTHLH
H13	TGT	0.1242	0.1204	0.2751	1	0.5999	rs10492364|rs6248|rs6245	PTHLH
H13	TGC	0.0222	0.02288	0.03872	1	0.844	rs10492364|rs6248|rs6245	PTHLH
H13	TAC	0.1838	0.1911	0.6954	1	0.4043	rs10492364|rs6248|rs6245	PTHLH
H13	CAC	0.6697	0.6656	0.1531	1	0.6956	rs10492364|rs6248|rs6245	PTHLH
H14	OMNIBUS	NA	NA	3.058	2	0.2167	rs2796|rs805512	PTHLH
H14	TC	0.149	0.1548	0.5246	1	0.4689	rs2796|rs805512	PTHLH
H14	CC	0.1817	0.1675	2.896	1	0.08878	rs2796|rs805512	PTHLH
H14	CA	0.6692	0.6777	0.6611	1	0.4162	rs2796|rs805512	PTHLH
H15	OMNIBUS	NA	NA	2.249	2	0.3248	rs997199|rs997200	PTHLH
H15	AC	0.1554	0.1473	1.032	1	0.3096	rs997199|rs997200	PTHLH
H15	CC	0.2766	0.29	1.778	1	0.1824	rs997199|rs997200	PTHLH
H15	CT	0.568	0.5627	0.2347	1	0.6281	rs997199|rs997200	PTHLH
H16	OMNIBUS	NA	NA	1.48	2	0.4772	rs3794275|rs27633|rs39635	PTHLH
H16	AGA	0.1372	0.1292	1.141	1	0.2854	rs3794275|rs27633|rs39635	PTHLH
H16	CGA	0.2044	0.2115	0.6145	1	0.4331	rs3794275|rs27633|rs39635	PTHLH
H16	CTC	0.6584	0.6593	0.00786	1	0.9294	rs3794275|rs27633|rs39635	PTHLH
**H17**	**OMNIBUS**	**NA**	**NA**	**27.05**	**3**	**5.75E-06**	**rs12425376|rs10843047|rs42294**	**PTHLH**
H17	TGG	0.2386	0.2461	0.6053	1	0.4366	rs12425376|rs10843047|rs42294	PTHLH
H17	TGT	0.0194	0.00747	26.77	1	2.30E-07	rs12425376|rs10843047|rs42294	PTHLH
H17	TAT	0.1736	0.1726	0.01355	1	0.9073	rs12425376|rs10843047|rs42294	PTHLH
H17	CAT	0.5684	0.5739	0.2481	1	0.6184	rs12425376|rs10843047|rs42294	PTHLH
H18	OMNIBUS	NA	NA	0.9755	2	0.614	rs11049257|rs33083	PTHLH
H18	CA	0.1497	0.1425	0.8541	1	0.3554	rs11049257|rs33083	PTHLH
H18	TA	0.1131	0.1117	0.04398	1	0.8339	rs11049257|rs33083	PTHLH
H18	TG	0.7372	0.7459	0.8019	1	0.3705	rs11049257|rs33083	PTHLH

Significant SNPs were highlighted in bold.

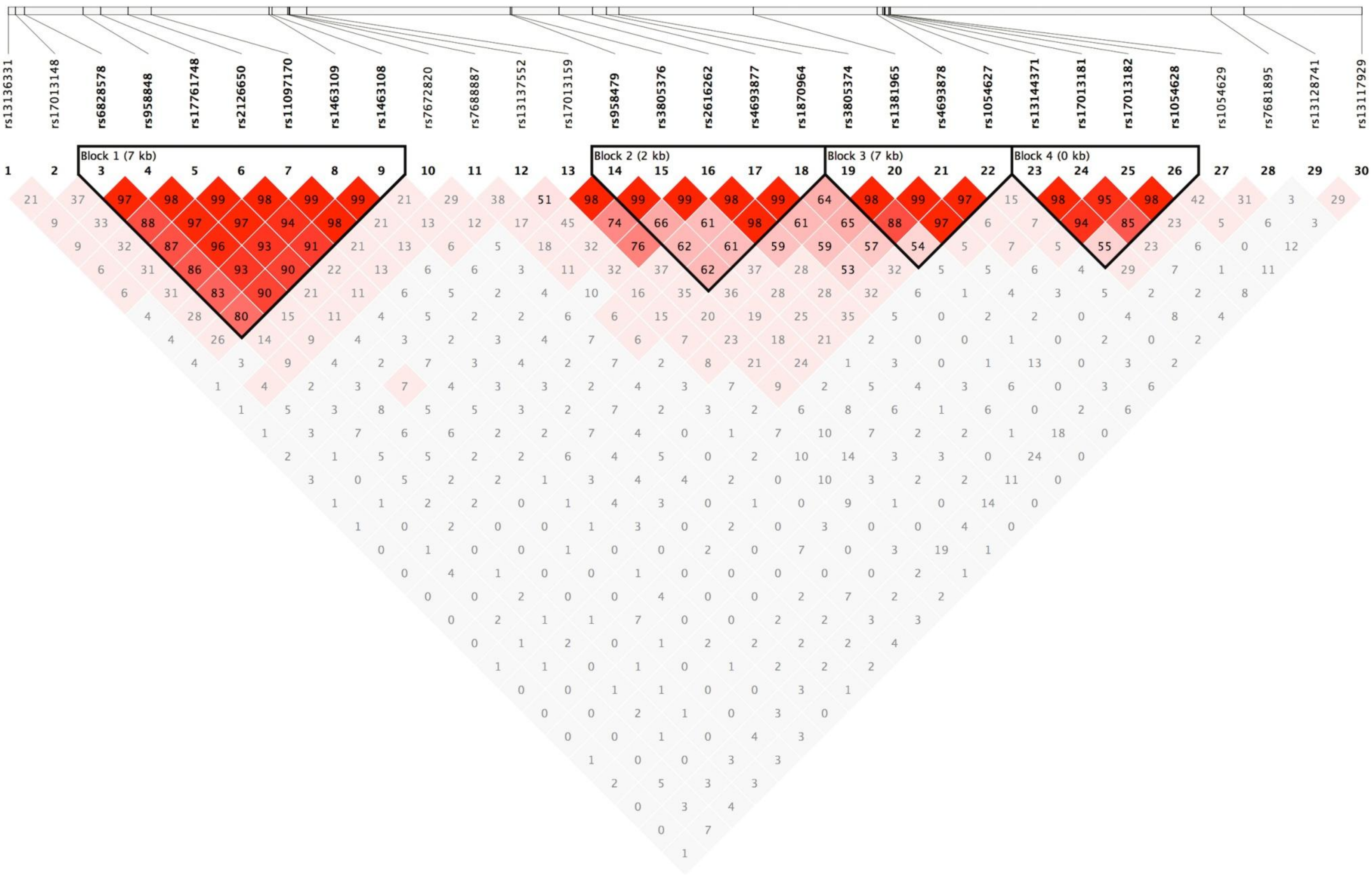

Supplemental Figure S1. Estimation of LD between each pair of 30 SNPs genotyped in *IBSP* gene in Han Chinese population. LD structure (D’) between marker pairs was indicated by the shaded matrices.

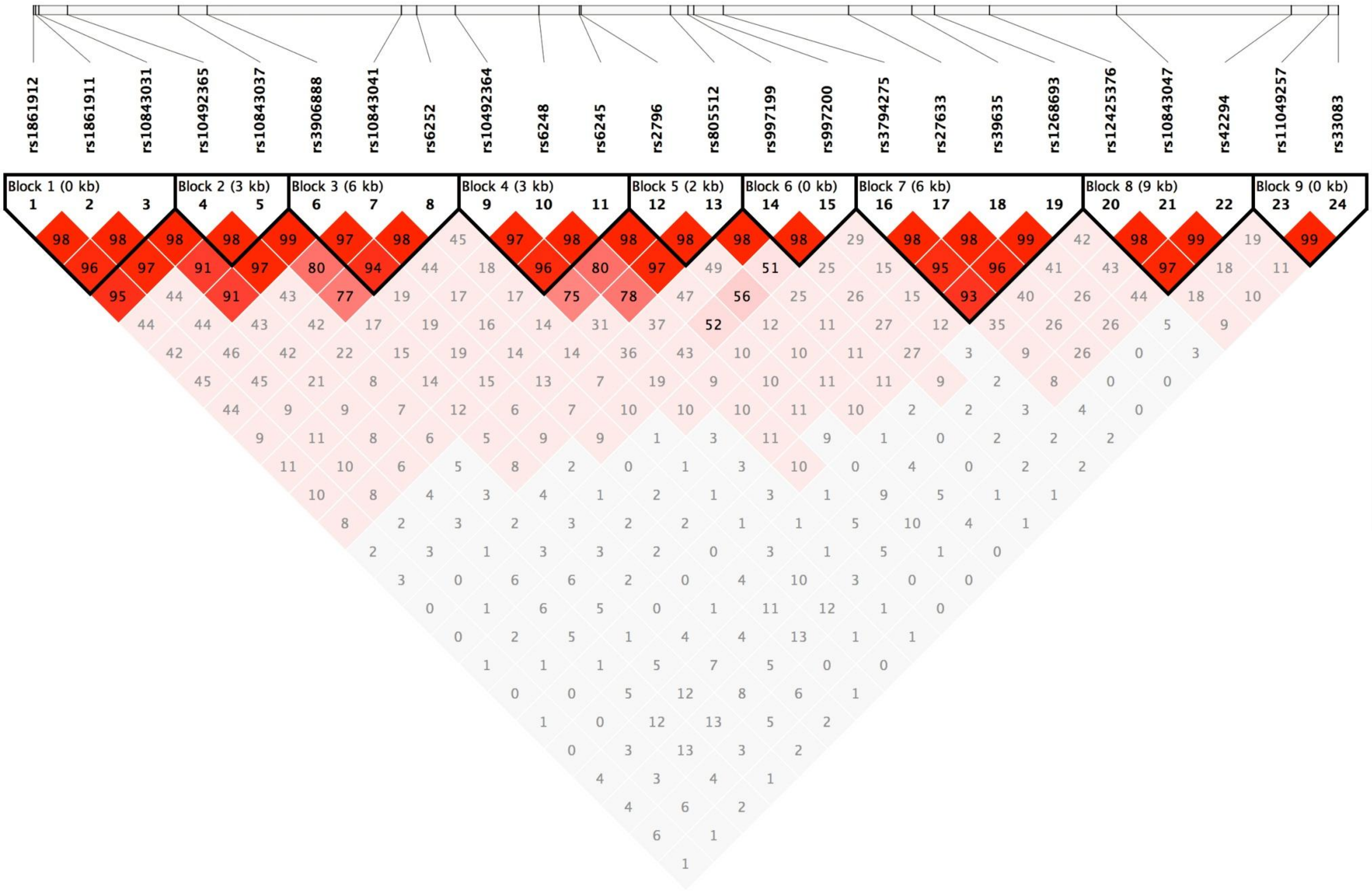

Supplemental Figure S2. Estimation of LD between each pair of 24 SNPs genotyped in *PTHLH* gene in Han Chinese population. LD structure (D') between marker pairs was indicated by the shaded matrices.
